# Dynamics of CD11c^+^ dendritic cell subsets in lymph nodes draining the site of intestinal nematode infection

**DOI:** 10.1016/j.imlet.2009.09.001

**Published:** 2009-12-02

**Authors:** Adam Balic, Katherine A. Smith, Yvonne Harcus, Rick M. Maizels

**Affiliations:** Institute of Immunology and Infection Research, University of Edinburgh, West Mains Road, Edinburgh EH9 3JT, UK

**Keywords:** Cytokines, Helminth infection, Migration, Mucosal immunity

## Abstract

Helminth parasites drive dominant Th2 responses through an as yet unidentified pathway. We have previously shown that the rodent gastrointestinal nematode *Nippostrongylus brasiliensis* secretes products which selectively activate *in vitro*-derived dendritic cells to induce Th2 responses on *in vivo* transfer. We now show that, during active infection with this parasite, the draining mesenteric lymph node dendritic cell population is altered significantly. Although there is substantial expansion of DC numbers during infection, the CD86^hi^-CD8α^int^-CD11b^−^ subset is markedly diminished, and expression levels of CD40, CD86 and CD103 are reduced. Notably, the reduced frequency of CD8α^int^ DCs is evident only in those mesenteric lymph nodes draining the anterior site of infestation. In infections with the longer lived *Heligmosomoides polygyrus*, the proportion of CD8α^int^ DCs in the MLNC falls to below 10% of total DC numbers by 35 days post-infection. Further, infection alters TLR responsiveness, as IL-12 production (as measured by *ex vivo* intracellular staining of CD11c^+^ DCs) in response to LPS stimulation is reduced, while IL-6, TNF-α and in particular, IL-10 all increase following infection with either nematode parasite. These changes suggest the possibility that helminth parasites modulate gastrointestinal immunity both by inhibiting migration of CD8α^int^ DCs to the draining lymph nodes, and modifying DC responsiveness in a manner which favours a Th2 outcome.

## 1 Introduction

Intestinal nematode infections are extraordinarily prevalent in the global human population [1,2] and are, like other helminth parasitisms, closely associated with Th2-dominated immune responses [3–7]. While Th2-mediated mechanisms can often cause expulsion of intestinal helminths, the precise pathways differ from one species to another, presumably because each parasite has evolved a unique evasion strategy which is optimally adapted to a particular niche within the host [8]. In mice, for example, *Nippostrongylus brasiliensis* is a potent stimulator of Th2-skewed immunity [9–11], and mice are able to expel this parasite through an IL-4/IL-13, STAT-6-dependent pathway involving intestinal goblet cell proliferation and mucus production [12]. In contrast, *Heligmosomoides polygyrus* elicits a combination of Th2 and Treg responses [7,13–15] which result in chronic infection, and only after drug-induced clearance is immunity to re-infection apparent, mediated by IL-4R-dependent alternatively activated macrophages [8,16].

Immune responsiveness to parasite infection is critically influenced by dendritic cell (DC) recognition and activation following encounter with invading organisms [17]. For example, DC-derived IL-12 is an essential initiating factor for the protective Th1 response in *Toxoplasma gondii* protozoal infections [18]. However, there is relatively little information on the profile of dendritic cells *in vivo* following exposure to nematode parasites. We have previously demonstrated that DCs exposed *in vitro* to antigens from *N. brasiliensis* show suppressed IL-12p70 responsiveness and elicit, on transfer to a naïve host, a dominant Th2 response [19]. Similar experiments with products from other helminths show that IL-12p70 down-regulation may be a common feature of the host immune response to metazoan parasites [20–22]. Hence, Th2 responsiveness may represent a programmed reaction of DCs to helminth pathogen-associated molecular patterns, in order to promote potentially protective Th2-associated immune mechanisms. However, it is not yet known whether the DC populations *in situ* during a helminth infection are indeed actively promoting Th2 responsiveness or if (for example) granulocyte or other populations are acting in a decisive way [23].

The gastrointestinal tract is the most extensive mucosal interface in the mammalian body, and is richly endowed with specialised DC populations [24–26]. CD11c^+^ DCs are widely distributed across the lamina propria (LP, underlying the epithelial layer in the intestine), Peyer's Patches (PP, lymphoid tissue directly attached to the gut) and a longitudinal series of mesenteric lymph nodes (MLN, which drain the intestinal tissues), with each locale comprising distinctive profiles of DC populations. LP DC comprises two phenotypes (myeloid-like CD11b^hi^CD8α^−^ and lymphoid-like CD11b^lo^CD8α^int^); the latter reported to be an LP-specific subset [27,28] which migrate to the MLN in a CCR7-dependent manner [28]. The PP also include myeloid and lymphoid DCs, although the latter are CD8α^hi^, as well as double-negative (DN) CD11c^+^ DCs lacking both CD11b or CD8α [27,29,30]. Within the MLN, each of these populations can be found in a steady-state [31]; however, the influx of CD8α^int^ DCs from the LP is greatly expanded following generic adjuvant-aided stimulation, for example with cholera toxin [32]. Most recently, attention has been drawn to the CD103-expressing subset of mucosal DCs, which induce T cells to express trafficking receptors for entry into gut tissues [33], produce retinoic acid which favours regulatory T cell induction [34] and, in contrast to CD103^−^ DCs, are able to take up orally delivered antigens for presentation to both CD4^+^ and CD8^+^ T cells [35].

It is known that the mesenteric lymph node is a critical locale for DC–T cell interactions [36,37], so that DC populations found in this tissue are likely to be of paramount importance to the outcome of intestinal nematode infections. We therefore investigated changes to MLN DCs following infection with *N. brasiliensis* and *H. polygyrus*, with respect to both the phenotypic subsets present over time, and the functional capacity of these DCs to produce cytokines known to be important in determining the Th1/Th2 balance of the immune response.

## 2 Materials and methods

### 2.1 Mice and parasites

BALB/c and C57BL/6 mice, 6–8 weeks of age, were bred in-house and maintained according to Home Office guidelines. *N. brasiliensis* parasites were maintained as previously described [9,38]. Infections were performed with 300 L3 s.c. and lymph nodes and spleens recovered 7 days later. *H. polygyrus* parasites were maintained as previously described [13]; mice were infected with 200 L3 by oral gavage, and lymph nodes and spleens recovered 1–5 weeks later.

### 2.2 DC purification

Mesenteric LNs, popliteal LNs, spleen and Peyer's Patches were recovered and placed in 5 ml of RPMI1640 (Gibco) and mechanically disrupted with scalpel blades in the presence of 0.1 mg/ml DNase I (Sigma). This preparation was then passed through a cell strainer to make single cell suspensions. Enrichment for DC populations was then performed by positive selection on Miltentyi Biotec CD11c-MACS beads. For experiments testing cytokine responsiveness to TLR ligation, spleens and lymph nodes were treated with liberase CI for 25 min and EDTA for 5 min before homogenisation and positive selection on CD11c MACS beads. Typically magnetic sorting produced a population at least 80% CD11c^+^.

### 2.3 Analysis of DC surface markers and intracellular cytokines

Expression of DC surface markers after purification was determined by flow cytometry using FITC-conjugated anti-CD11c, PE-conjugated anti-CD8α, anti-CD40, anti-CD80, anti-CD86, anti-MHC-II or biotin-conjugated anti-CD8α, anti-CD11b, anti-CD103, anti-CD295 (DEC205), anti-B220 mAbs, followed by APC-strepavidin staining. All mAb were purchased from BD PharMingen. Samples were analyzed using a FACSCalibur flow cytometer and FlowJo software package (Tree Star). Intracellular cytokines were detected by stimulating DC with 1 μg/ml LPS as described previously [19] for 6 h in the presence of 20 μg/ml Brefeldin A (Sigma, UK). After 6 h the cells were stained for surface CD11c as described above, fixed using a Cytofix/Cytoperm PlusTM kit (PharMingen) and intracellular IL-6, IL-12 or TNF-α detected using PE-conjugated mAb and the PharMingen Cytofix/Cytoperm kit as per manufacturer's instructions. After staining cells were analysed by flow cytometry using a FACSCalibur flow cytometer and FlowJo software. DCs were identified as CD11c^+^, viable cells.

### 2.4 ELISA

Cytokines released into culture supernatants were measured by ELISA using capture/detection combinations specific for IL-6, IL-10 and TNF-α as previously described [19]. Cytokine concentrations were determined by reference to a standard curve of these mediators.

## 3 Results

### 3.1 DC subsets in the mesenteric lymph nodes

**Fig. 1:**
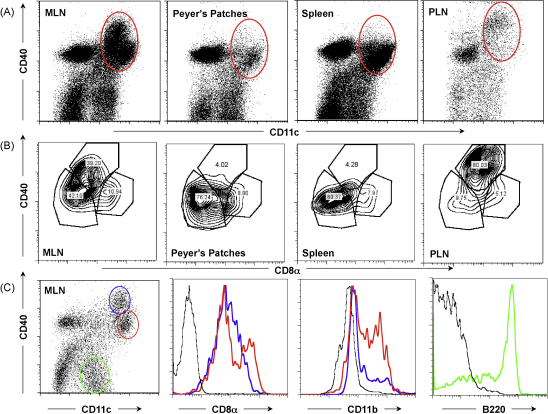
DC subsets in the mesenteric lymph node and other lymphoid tissues. (A) CD11c^+^ MACS column-enriched DC populations from naïve mesenteric lymph node (MLN), Peyer's Patches, spleen and popliteal lymph node (PLN) were stained with anti-CD40 and anti-CD11c. Red ovals delineate lymphoid/myeloid DC populations analyzed in (B) below. (B) CD11c^+^ DC subpopulations analyzed for CD8a and CD40 from the same tissues. Note the presence of CD8α intermediate (CD8α^int^) CD40 high (CD40^hi^) populations only in the lymph nodes. (C) Subpopulations of MLN CD11c^+^ DCs, as shown in (A) above, gated as shown in left panel, representing predominantly lymphoid DCs (blue), myeloid DCs (red) and plasmacytoid DCs (green). Gated populations were analysed for CD8α and CD11b (blue and red gates), and for B220 (green gate).

Subpopulations of DCs in the mesenteric lymph nodes (MLNs) of mice are known to share many properties with, and show some differences from, DCs in other immunological tissues [27,28,31]. We first established the profile of MLN DCs within our colony of BALB/c mice, in comparison to spleen, popliteal lymph node, and Peyer's Patches, enriching for CD11c^+^ populations by magnetic bead sorting. As shown in Fig. 1A, the lymph nodes are relatively rich in cells expressing high levels of CD40, which are infrequent in spleen and PP. Moreover, within the CD11c^+^CD40^+^ population from each lymphoid organ (circled in Fig. 1A), a major CD8α^int^CD40^hi^ subset is present only in the lymph nodes (Fig. 1B). Further analysis within the MLN population, gating by CD40 expression (Fig. 1C), confirms that the CD40^hi^ subset are largely lymphoid in type, being CD8α^int^ and CD11b^−^; this subset is also B220^−^ (data not shown). Myeloid DCs, expressing CD11b, are mostly found in the CD40-intermediate gate. In contrast the CD40^−^ population show a B220^+^ plasmacytoid phenotype.

**Fig. 2:**
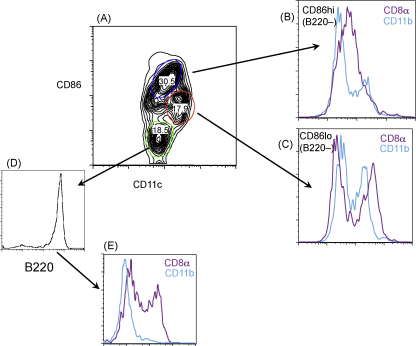
DCs expressing high levels of CD86 are primarily CD8α^int^ lymphoid DCs. (A) CD11c^+^ MACS column-enriched DCs were stained with anti-CD86 and CD11c, and gated according to high (blue oval), intermediate (red oval) or low (green oval) CD86 expression. (B) Co-staining with anti-CD8α, CD11b shows that the CD86^hi^ subset are largely CD8α^int^CD11b^−^ lymphoid DCs. (C) Co-staining with anti-CD8α, CD11b shows that the CD86^int^ population combines myeloid (CD11b^+^) and CD8α^hi^ lymphoid cells; both populations are B220^−^ (not shown). (D) CD86^−^ cells are uniformly B220^+^. (E) CD86^−^ cells are CD11b^−^, but are heterogeneous with respect to CD8a expression.

MLN DCs were also analysed according to their levels of CD86 expression (Fig. 2A). The CD86^hi^ population predominantly displayed a CD8α^int^ phenotype, and this population was largely CD11b^−^ (Fig. 2B). The CD86^int^ population, which were CD11c^hi^, comprised a mixture of CD8α^hi^ and CD8α^−^ (Fig. 2C). In contrast, the CD86 low/negative cell subset were plasmacytoid in character, being B220^hi^ (Fig. 2D) and CD11b^−^ (Fig. 2E). This latter subset divides evenly into CD8α^−^ and CD8^hi^ expressing cells.

### 3.2 Response to *N. brasiliensis* infection

**Fig. 3:**
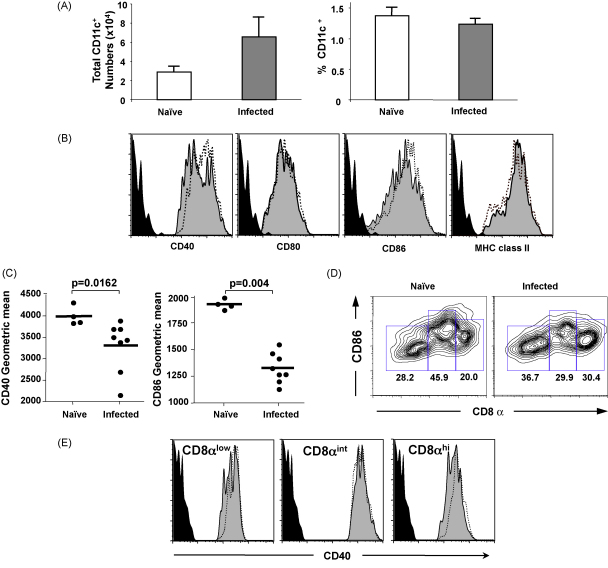
MLN DCs following 7 days of *Nippostrongylus brasiliensis* infection. (A) Expansion of lymph node cell numbers during infection; left panel shows total CD11c^+^ numbers in MLNs from naïve mice and from mice taken 7 days following *N. brasiliensis* infection. Right panel shows percentage of CD11c^+^ cells within total MLN populations in each group of mice. (B) CD11c^+^ DCs following *N. brasiliensis* infection (grey fill) show diminished CD40 and CD86 expression, but unaltered CD80 or MHC class II expression. (C) Differences in geometric mean fluorescence intensity for CD40 and CD86 staining between MLN CD11c^+^ DCs from naïve mice and from mice taken 7 days following *N. brasiliensis* infection. (D) Frequency of CD8^int^CD86^hi^ cells is reduced following *N. brasiliensis* infection. (E) Within the CD8 subpopulations, *N. brasiliensis* infection (grey fill), has a differential effect on peak CD40 expression.

*N. brasiliensis* causes a short-lived infection of the anterior small intestine, commencing 48 h after entry of skin-penetrating larvae into rodents, and concluding with the immune-mediated expulsion of adult worms 5–7 days following infection. During this time, we noted that the MLN expands 2–3-fold in cell numbers, within which a constant proportion of DCs is maintained (Fig. 3A). Although CD80 and MHCII remained unaltered, there was a clear diminution in CD40 and CD86 expression levels within the CD11c^+^ DC population with fewer high-expressing CD40 and CD86 cells (Fig. 3B). This loss was reflected in significant reductions in intensity of surface staining for these two markers on DCs from infected mice (Fig. 3C). Interestingly, the reduction in CD86 could be accounted for by a paucity of CD86^hi^CD8α^int^ cells, which are relatively diminished following infection (Fig. 3D). In the case of CD40, a downshift in expression was detectable on CD8α^lo^ and CD8α^hi^ populations (Fig. 3E), which in combination with their higher frequency following infection (Fig. 3D) and their lower consitutive levels of CD40 (Fig. 1), reduces the overall intensity of this marker within the MLN DC population. As MLNs expand at least twofold within 7 days of infection, the diminution of CD8α^int^ DCs could reflect either outgrowth by other cell types, or poor recruitment of this subset from the lamina propria.

### 3.3 Changes to DC populations are related to the site of infection

**Fig. 4:**
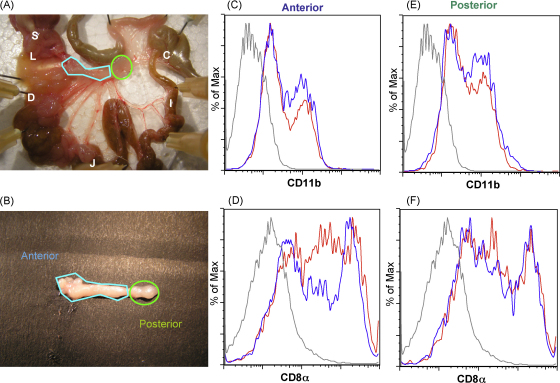
Reduction in CD8α^int^ frequency is localised to nodes draining the site of infection. (A) Gut of BALB/c naïve mouse. Blue polygon = anterior nodes, Green oval = posterior nodes, S = stomach, L = liver, D = duodenum, J = jejuenum, I = ileum, C = caecum. (B) Lymph nodes dissected out from the same specimen. (C–F) Naïve (red) and *N. brasiliensis*-infected (blue) MLN DC were recovered separately from anterior and posterior nodes. Note decrease in CD8α^int^ DC only in the anterior nodes, which drain the site of infestation. Grey line shows isotype control.

Because *N. brasiliensis* infests the anterior part of the small intestine, we compared the changes in DC subsets in the anterior and posterior lymph nodes, as defined in Fig. 4A. It is important to note that the longitudinal organisation of the gut lymphoid tissue involves a series of mesenteric lymph nodes which each drain successive sections of the gastrointestinal tract. We isolated DCs from the anterior and mid-sections of the MLNs, draining the site of infection, and compared them to the posterior LNs, which are also exposed to a higher level of bacterial colonisation (Fig. 4B). Expression of CD11b was broadly similar in anterior and posterior LNs, and was not greatly altered by infection (Fig. 4C, E). However, CD8α^int^ DCs were more prominent in anterior MLNs, and this phenotypic subset showed the greatest difference between naïve and infected mice. Hence, the reduction in CD8α^int^ cells was observed only in the nodes directly draining the site of infection, and not in the more posterior sites.

### 3.4 Reductions in CD103 and CD205 accompany loss of CD8α^int^ DCs

**Fig. 5:**
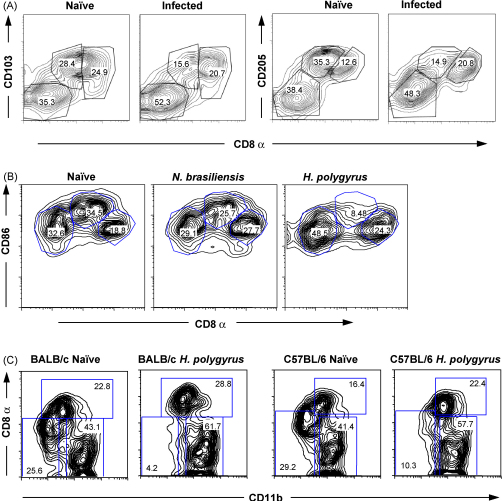
Effect of infections on the distribution of DC subpopulations. (A) CD103 and CD205 (DEC205) expression in CD11c^+^ DCs, plotted against CD8α expression Numbers represent percentage of total cells within the indicated gates. (B) Comparative effect of *N. brasiliensis* and *Heligmosomoides polygyrus* infection on CD11c^+^ DC CD8α, CD86 expression. MLN DCs were analysed at 7 and 35 days of infection, respectively. (C) Strain-independence of loss of CD8α^int^ in chronic *H. polygyrus* infection.

The diminished presence of CD8α^int^ DCs in infection had further consequences for the expression of a range of important surface molecules among mucosal DCs. In naïve MLN, CD8α^int^ cells express the highest levels of CD103, and overall levels are reduced within 7 days of *N. brasiliensis* infection (Fig. 5A). Similar reductions are seen in CD205 (DEC-205, Fig. 5B) as well as L-selectin (data not shown).

To investigate whether the relative loss of CD8α^int^ cells over 7 days would accentuate in a longer term nematode infection, we also studied mice carrying chronic *H. polygyrus* infection. At 5 weeks post-infection, when the duodenum is chronically infected with this parasite, the MLN show an almost complete absence of CD8α^int^ (Fig. 5B), effectively removing the highest CD86-expressing cells from the MLN (data not shown). The loss of lymphoid CD8α^int^ DCs was also found to be equally profound in BALB/c and C57BL/6 strains of mice (Fig. 5C).

### 3.5 Altered cytokine responses to TLR stimulation in infection

**Fig. 6:**
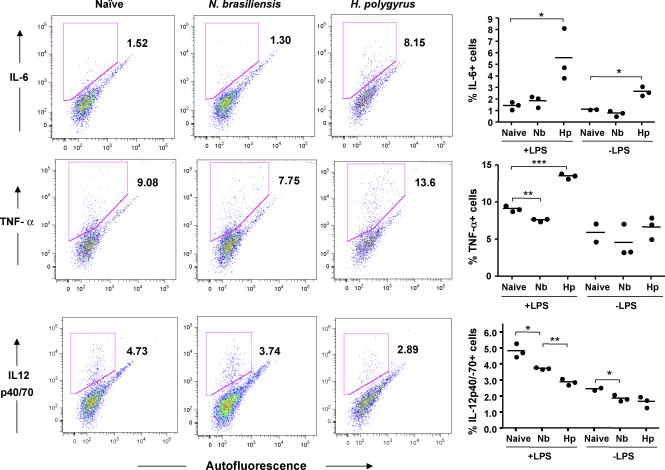
Intracellular cytokine responses of DC populations to TLR ligation. MLN from C57BL/6 mice were taken 7 days following infection with *N. brasiliensis* (Nb) or *H. polygyrus* (Hp). Organs were harvested, treated with liberase CI for 25 min and EDTA for 5 min, then homogenised and labelled with CD11c microbeads (Miltenyi) before selecting on a LS column. Following selection, CD11c^+^ cells were counted, plated at 10^5^/well with 1 μg/ml LPS and 20 μg/ml brefeldin A for 6 h. Cells were then harvested and stained intracellularly with PE-conjugated IL-6, TNF-α, IL-12p40/70, or an isotype control (IgG1). Left hand panels show representative flow cytometry plots of intracellular staining against autofluorescence measured in the FITC channel, for LPS-stimulated DCs; right hand panels show data from individual mice, and include unstimulated control cells cultured in the absence of LPS. ^*^p < 0.05, ^**^p < 0.01, ^***^p < 0.001. Data presented are representative of two experiments with similar results.

To assess the functional properties of MLN DCs from uninfected and infected mice, MACS-enriched CD11c^+^ DCs were challenged *in vitro* with the TLR4 ligand LPS, and subsequently stained for intracellular expression of IL-6, IL-12 and TNF-α (Fig. 6). IL-12 responses were reduced in DCs from mice infected with either *N. brasiliensis* or *H. polygyrus*. In the case of DCs from *H. polygyrus*-infected mice, there was a marked enhancement of IL-6 and TNF-α responsiveness (Fig. 6).

**Fig. 7:**
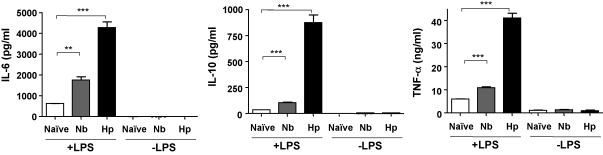
Cytokine release by DCs from naïve and infected MLNs. Spleens and MLN from C57BL/6 mice were taken 7 days following infection with *N. brasiliensis* (Nb) or *H. polygyrus* (Hp), and CD11c^+^ DCs isolated as described in the legend to Fig. 6. CD11c^+^ cells, pooled from 4 to 6 mice per group, were plated in triplicate at 10^4^/well with or without 1 μg/ml LPS and supernatants harvested 72 h later before analysing by ELISA. Similar results, but lower magnitude responses, were obtained from parallel cultures of splenic DCs (data not shown). Data presented are representative of two experiments with similar results. ^**^p < 0.01; ^***^p < 0.001.

In further experiments, cytokines released into the culture supernatants were measured. DCs from mice infected with either nematode parasite secreted significantly more IL-6, IL-10 and TNF-α into the medium, with the largest relative increase observed for secretion of IL-10 (Fig. 7). Similar trends were seen in both MLN and spleen (data not shown), but the magnitude of the effect was larger and reached higher levels of significance in the MLN samples. Interestingly, inhibition of DC IL-12 production and enhancement of IL-6 responses has previously been reported for stimulation of bone marrow-derived murine DCs by the secreted protein fraction of *N. brasiliensis* [19], while *H. polygyrus* secretions have also been shown to inhibit TLR-dependent IL-12 responses of murine DCs [21].

## 4 Discussion

The intestinal environment is remarkable for its ability to maintain homeostasis in the face of abundant foreign antigenic material in the form of food proteins and commensal bacteria, via the induction of oral tolerance. At the same time, the immune system must remain alert to the entry of pathogens, and sentinel DCs in the intestine retain the capacity to stimulate strong immune responses when this may be appropriate [24,39]. The ingress of parasitic helminths represents an interesting, and unexplored, challenge to the discriminatory powers of the immune system. We report here that the MLN DC population responds quickly to helminth infection, with marked changes in subset distribution and capacity to produce cytokines in response to stimulation.

A central and fascinating question in mucosal immunology revolves around the function and interrelationship of DC subpopulations in the gastrointestinal tract [25,26,40]. While most DCs conform to stereotypical myeloid (CD11b^+^CD8α^−^), lymphoid (CD11b^−^CD8α^+^) or plasmacytoid (B220^+^) subsets [41,42], the mesenteric lymph nodes contain a lamina propria-derived migratory DC population described as CD8α^int^ [27,28,32,43]. In contrast, the CD8α^−^ and CD8α^hi^ DC subpopulations in the MLN may represent DC recruited from the blood [32,43] or PP [28], or may arise by local proliferation in the LN [35].

Recently oral tolerance to soluble antigens has been shown to be due to the migration of LP DC to the MLN via the afferent lymphatics not due to contributions from the PP or other gut associated secondary lymphoid tissue [37]. CCR7 deficient mice have normal numbers of DC in the LP, but dramatically reduced numbers of all DC subpopulations in the MLN [37]. Therefore, our finding that the CD8α^int^ type is, in relative terms, depleted in nematode infection is of particular interest: firstly, it implies that parasites may interfere specifically with the migratory potential of lamina propria DCs; and secondly, these data indicate that the CD8α^int^ phenotype is a distinct population rather than a low-expressing variant of the conventional lymphoid subset.

The possibility that CD8α^int^ DCs convert into another phenotype over time, or according to their tissue environment, is not supported by experimental data. Experiments that have tracked peripheral antigen uptake to the local draining lymph node have demonstrated that the antigen containing DC are CD8α^int^, with no labelling of the CD8α^hi^ subpopulation [43], suggesting that CD8α^int^ cells do not convert to a CD8α^hi^ phenotype, at least while ingested antigen is detectable. Moreover, administration of oral adjuvant which causes a large increase in CD8α^int^ DC emigration from the gut to the MLN does not result in a subsequent increase in CD8α^hi^ DC [32], again suggesting that the CD8α^int^ subpopulation is not converting into the CD8α^hi^ population. Conversely, infection with mouse mammary tumour virus which blocks migration of DC from the skin, results in a massive increase in the absolute number of blood-borne CD8α^−^ and CD8α^hi^ DC in the local draining lymph node with no effect on CD8α^int^ numbers [43]. Taken together, these data suggest that during steady state and infection, CD8α^int^ and CD8α^hi^ DC subpopulations are independent.

Tissue-derived migratory DCs have also been characterised by expression of surfaces markers such as CD103 [35] and CD205 [44]. In the intestinal setting, CD103^+^ DCs migrate from the lamina propria (LP) to the MLNs in a CCR7-dependent manner [28]; this DC subset produces retinoic acid and promotes the generation of Foxp3^+^ regulatory T cells [34]. Because in both *N. brasiliensis* and *H. polygyrus* infections, there is relative decline of the CD8α^int^ subpopulation which express the highest levels of CD103 and CD205, it appears that the DC response in the MLN to gastrointestinal nematode parasites is dominated by an increase in resident lymphoid DC subpopulations, and not by increased recruitment of DC from the site of infection.

More generally, we have shown that MLN DCs from infected mice are functionally shifted away from Th1-type cytokine production (in terms of IL-12) and towards a Th2/regulatory profile with enhanced IL-10 release *in vitro*. In similar experiments, it has been reported that DCs from *H. polygyrus*-infected mice (taken as a pool of spleen and MLNC) were able to inhibit the Th1 response to bacterial infection on adoptive transfer [45]. Although these authors did not describe the CD8 expression profile, their cells correspond closely to those we show here that lack the CD8α^int^ population (Fig. 5C).

The inflammatory response in the gut during *N. brasiliensis* has been reported to be localised to the site of infection [46]. In the present study a reduction in tissue-derived migratory CD8α^int^ was confined to the anterior MLN, which drain the anterior duodenal site of infestation. Thus, changes in DC migration from the small intestine were localised in a linear fashion even within the small intestine. This in turn suggests either that parasite antigens do not reach the posterior small intestine, or that the change in DC population dynamics we report are a consequence both of antigens secreted by the parasite, and of the inflammatory response associated with parasite feeding on the mucosa.

Importantly, in different helminth contexts, the suppression of IL-12 production appears to be a common theme [19–22] which may be particularly significant in terms of mitigating inflammation caused by bacterial exposure in invaded gut tissue. Because commensal bacteria are sparse in the stomach, duodenum and jejunum, and progressively more abundant through the distal ileum and colon, there may be contrasting microbiological influences on different nodes, and this may well underlie the regionalisation of both steady-state DC populations, and their response to infection, as noted above.

Tissue-derived migratory DCs have a central function in antigen presentation of orally delivered soluble antigen and the maintenance of immunological tolerance. Interestingly, this requires migration from the LP to the MLN, while the failure of CD8α^int^ DCs to expand in number implies that this trafficking is inhibited in gastrointestinal nematode infection. Since delivery of commensal antigen to MLN is considered to be essential for oral tolerance [37], it is possible that infection interrupts oral tolerance thereby explaining a long-standing observation in *N. brasiliensis* [47]. Possibly, the changes in DC dynamics favour the evolution of a Th2 response in the gut, which in the case of *N. brasiliensis* can expel the parasite within 5–7 days of infection. However, an unanswered question is whether the DC populations present after a longer period of chronic infection (28 days in the case of *H. polygyrus*) are involved in the generation of regulatory T cells, which in this infection are known to be expanded in number and in functional capacity [13–15]. As oral tolerance to soluble antigen requires the migration of LP DC to the MLN and conversely chronic *H. polygyrus* infection may block migration of LP DC to the MLN, it implies that the mechanism(s) by which regulatory T cells are induced during chronic nematode infection have different requirements than the induction of oral tolerance. In future work, a broader set of potential APC populations will need to be assessed for regulatory T cell induction, including plasmacytoid DCs [48], B cells and alternatively activated macrophages [49]. These studies are now under way in our laboratory.
